# Recombinant annexin A2 inhibits peripheral leukocyte activation and brain infiltration after traumatic brain injury

**DOI:** 10.1186/s12974-021-02219-7

**Published:** 2021-08-09

**Authors:** Ning Liu, Jinrui Han, Yadan Li, Yinghua Jiang, Samuel X. Shi, Josephine Lok, Michael Whalen, Aaron S. Dumont, Xiaoying Wang

**Affiliations:** 1 Clinical Neuroscience Research Center, Department of Neurosurgery and Neurology, Tulane University School of Medicine, New Orleans, LA 70122 USA; 2 Neuroprotection Research Laboratory, Department of Radiology and Neurology, Massachusetts General Hospital, Harvard Medical School, Boston, MA 02129 USA; 3 Department of Pediatrics, Pediatric Critical Care Medicine, Massachusetts General Hospital, Harvard Medical School, Charlestown, MA 02129 USA

**Keywords:** Traumatic brain injury, Recombinant annexin A2, TLR4, Leukocyte infiltration, Neuroinflammation, Neurobehavioral

## Abstract

**Background:**

Traumatic brain injury (TBI) is a significant cause of death and disability worldwide. The TLR4-NFκB signaling cascade is the critical pro-inflammatory activation pathway of leukocytes after TBI, and modulating this signaling cascade may be an effective therapeutic target for treating TBI. Previous studies indicate that recombinant annexin A2 (rA2) might be an interactive molecule modulating the TLR4-NFκB signaling; however, the role of rA2 in regulating this signaling pathway in leukocytes after TBI and its subsequent effects have not been investigated.

**Methods:**

C57BL/6 mice were subjected to TBI and randomly divided into groups that received intraperitoneal rA2 or vehicle at 2 h after TBI. The peripheral leukocyte activation and infiltrating immune cells were examined by flow cytometry, RT-qPCR, and immunostaining. The neutrophilic TLR4 expression on the cell membrane was examined by flow cytometry and confocal microscope, and the interaction of annexin A2 with TLR4 was assessed by co-immunoprecipitation coupled with Western blotting. Neuroinflammation was measured via cytokine proteome profiler array and RT-qPCR. Neurodegeneration was determined by Western blotting and immunostaining. Neurobehavioral assessments were used to monitor motor and cognitive function. Brain tissue loss was assessed via MAP2 staining.

**Results:**

rA2 administration given at 2 h after TBI significantly attenuates neutrophil activation and brain infiltration at 24 h of TBI. In vivo and in vitro data show that rA2 binds to and reduces TLR4 expression on the neutrophil surface and suppresses TLR4/NFκB signaling pathway in neutrophils at 12 h after TBI. Furthermore, rA2 administration also reduces pro-inflammation of brain tissues within 24 h and neurodegeneration at 48 h after TBI. Lastly, rA2 improves long-term sensorimotor ability and cognitive function, and reduces brain tissue loss at 28 days after TBI.

**Conclusions:**

Systematic rA2 administration at 2 h after TBI significantly inhibits activation and brain infiltration of peripheral leukocytes, especially neutrophils at the acute phase. Consequently, rA2 reduces the detrimental brain pro-inflammation-associated neurodegeneration and ultimately ameliorates neurological deficits after TBI. The underlying molecular mechanism might be at least in part attributed to rA2 bindings to pro-inflammatory receptor TLR4 in peripheral leukocytes, thereby blocking NFκB signaling activation pathways following TBI.

**Supplementary Information:**

The online version contains supplementary material available at 10.1186/s12974-021-02219-7.

## Introduction

Traumatic brain injury (TBI) is a complex neurological injury. Emerging evidence suggests that neuroinflammation contributes to secondary brain injury following TBI. Activation of circulating immune cell-derived pro-inflammation plays a critical role in triggering neuroinflammation-associated secondary brain tissue damage at the acute phase following TBI [1]. Neutrophils are the most abundant circulating immune cell subset and the first group of peripheral blood leukocytes whose number increases dramatically within 48 h after TBI. Neutrophilic first responders to injury are rapidly recruited to the injured brain wherein they aggravate the secondary brain damage by amplifying the immune response of the injured brain [1–3]. This secondary injury worsens functional outcome and increases the mortality rate in patients with various degrees of TBI [3, 4]. Treatment options for TBI are restricted to symptomatic treatments; presently, there is still a large knowledge gap in understanding the detailed pathological mechanisms of early neutrophil activation and brain infiltration after TBI [5]. Emerging studies highlight the central role of the TLR4-NFκB signaling cascade in the pro-inflammatory activation of leukocytes after TBI [6, 7]. Therefore, modulating this signaling cascade may be an effective therapeutic target in TBI secondary injury.

Accumulating evidence suggests that cell membrane protein annexin A2 (AXNA2) can interact with neutrophils to modulate their inflammatory effect [8]. AXNA2 is a multi-compartmental protein expressed on various cells; it orchestrates multiple cellular processes, including fibrinolysis, exocytosis, endocytosis, membrane trafficking, and cell survival [9]. Through bulk RNA sequencing and multiple data-driven Bayesian network analysis, AXNA2 was identified as a critical driver of the genes responsible for regulating the cascade of cellular events involved in the TBI pathology [10]. Our recent study finds that AXNA2 deficiency exacerbates TBI-induced neutrophil infiltration, neuroinflammation, and long-term neurological outcome [11]. However, the mechanisms underlying the actions of AXNA2 in modulating TBI pathogenesis remain largely unknown. Interestingly, previous studies also document protein binding between endogenous AXNA2 and TLR4 in macrophages [12] and monocytes [13]. Moreover, it has been found the AXNA2 tetramer (A2t, consists of two S100A10 (p11) and two annexin A2) modulated macrophage function through TLR4, but not TLR2 [14]. Importantly, endogenous AXNA2 is capable of negatively regulating TLR4-triggered inflammatory responses in macrophages [12]. We therefore hypothesize that rA2 protein can inhibit the pro-inflammatory activation and brain infiltration of peripheral leukocytes by binding to TLR4 following TBI.

In the present study, we used the controlled cortical impact (CCI) mice model to test our hypotheses. We investigated the effects of rA2 administration on circulating leukocyte counts, activation, and brain infiltration, particularly for the neutrophils, as well as the involvement of TLR4/NFκB signaling pathway in these cells after TBI in mice. We further evaluated the effects of rA2 administration in the brain inflammatory profile, neurodegeneration, and neurological function deficits.

## Materials and methods

### Animals

Wild-type C57/BL6 mice (10–12 months old, 25–29 g) were purchased from the Jackson Laboratory. All animal studies were performed according to the protocol approved by the Tulane University School of Medicine under the National Institutes of Health Guide for Care and Use of Laboratory Animals. All experiments were performed with randomization, allocation concealment, and blinding.

### rA2 production and in vivo administration

rA2 was produced as we previously described [15]. Briefly, rA2 was expressed in an *Escherichia coli* (*E. coli*) induced by the isopropyl β-D-1-thiogalactopyranoside (IPTG), followed by *E. coli* collection, lysis, and centrifuge at 4 ℃ to obtain a supernatant. Subsequently, a combination of hydrophobic interaction chromatography, ion-exchange chromatography, and hydroxyapatite chromatography, which were produced in the Bioexpression and Fermentation Facility at the University of Georgia (http://bff.uga.edu/), was used to purify rA2. The final rA2 concentration is 8 mg/mL and the purity is 96% (endotoxin 0.5 EU/mg). A previous study had intrathecally (i.t.) injected AXNA2 with a dose of 100 μg/rat (250–300 g) into the spinal cord injury rat model [16]. Considering the effectiveness of intraperitoneal (i.p.) injection may be compromised compared to i.t., rA2 was i.p. into mice with the dose of 1 mg/kg. We determined the plasma concentrations of ANXA2 in C57BL/6 mice at 0, 2, 6, 12, 24, 48, and 72 h after single i.p. injection of rA2 (1 mg/kg) using enzyme-linked immunosorbent assay (ELISA). The peak of ANXA2 concentration (52 ± 14.58 ng/mg plasma) was observed at 2 h post-injection. The half-life was estimated to be 8.9 h (Supplemental Figure 1).

### Enzyme-linked immunosorbent assay

C57BL/6 mice were i.p. injected with rA2 (1 mg/kg), and blood were collected from the eye veins of the mice after anesthesia at 0, 2, 6, 12, 24, 48, and 72 h after injection. The blood samples were centrifuged at 2000 rpm for 15 min at 4 ℃ to collect the plasma. One hundred micrograms of plasma from each mice was used for enzyme-linked immunosorbent assay using the Human Total Annexin A2 DuoSet IC ELISA kit (Novus Biologicals, DYC3928-2) following the manufacturer’s protocol. Standard curves were generated using purified rA2. The absorbance was measured at 450 nm with a microplate reader (Molecular Devices). The plasma AXNA2 half-life was calculated using the following formula as described previously [17]: *t*_1/2_ = log 0.5/(log Ae/A0) × *t*, where *t*_1/2_ is the half-life, Ae is the amount of AXNA2 remaining, A0 is the amount of AXNA2 on 2 h, and *t* is the elapsed time.

### Controlled cortical impact model of traumatic brain injury in mice

The CCI model was used as we described previously [18, 19] with slight modification. Male C57BL/6 mice (10–12 months old, 25–29 g) were anesthetized with 2% isoflurane (Anaquest, Memphis, TN) in a closed plastic box for 3 min and maintained under 1.5% isoflurane with a face mask in 70% N_2_O and 30% O_2_ using a Fluotec 3 vaporizer (Colonial Medical Amherst, NH) in a stereotaxic apparatus. After skin disinfection using the povidone-iodine solution (7.5%), a skin incision was made to expose the skull and bregma. A 5-mm craniotomy was performed lateral to the midline, between bregma and lambda (over the left somatosensory cortex) using a portable trephine drill, and the skull flap was removed. Subsequently, a 3-mm flat-tipped impactor is placed on the dural surface, and a controlled cortical impact was carried out with 4.6 m/s impact velocity, 0.7 mm impact depth, and 500 ms impact dwell time on the TBI-0310 Impactor (Precision Systems and instrumentation, LLC). The incision is then closed by suture, and mice were placed in a clean cage to recover. The mice in the sham group were subjected to the same procedures but without impact.

### Flow cytometry

Flow cytometry was performed as previously described [20] with minor modifications. Single-cell suspensions were prepared from blood or brain tissues of mice at 24 h after TBI. Red blood cells were eliminated from blood samples using a lysing buffer (BD Biosciences) prior to antibody staining. Brain tissues were ground and homogenized with 70-mm nylon cell strainers in PBS. Thereafter, cell pellets were collected after centrifuging at 2000 rpm for 5 min and resuspended into 7 mL of 30% Percoll solution. After centrifuging at 700 g for 10 min at room temperature, cell pellets were collected for antibody staining. All antibodies were stained at 4 ℃ for 30 min following their instruction. For intracellular antigen staining, cells were fixed and permeated with a fixation/permeabilization solution kit (BD Biosciences). Phycoerythrin (PE), fluorescein isothiocyanate (FITC), allophycocyanin (APC), peridinin chlorophyll protein-cyanine 5.5 (PerCP-Cy5.5), eFluor450, APC-Cy7- or PE-Cy7-conjugated antibodies were purchased from BioLegend or eBioscience. The following antibodies to mouse clones were used: CD11b (Clone M1/70,48–0112-82, eBioscience), CD45 (Clone 30-F11,103,116, BioLegend), CD45 (Clone 30-F11,103,112, BioLegend), Ly-6G (Clone 1A8, 127,616, BioLegend), F4/80 (Clone BM8,123,116, BioLegend), F4/80 (Clone BM8,123,118, BioLegend), CD3 ε (Clone 145-2C11,100,312, BioLegend), CD3 ε (Clone 145-2C11,100,351, BioLegend), Ly-6C (Clone HK1.4, 128,016, BioLegend), CD69 (Clone H1.2F3,104,512, BioLegend), TLR4 (Clone UT41,12–9041-80, eBioscience), TLR2 (Clone 6C2, 50–100-57, eBioscience), TNFα (Clone TN3-19.12, 506,104, BioLegend), IL-6 (Clone MP5-20F3, 504,504, BioLegend), IL-10 (Clone JES5-16E3, 505,006, BioLegend). Cell surface phenotype and intracellular cytokine expression were performed on a BD LSRFortessa™ Cell Analyzer (BD Bioscience, San Jose, CA, USA). Data were analyzed with FlowJo software (Version 7.6.1, FlowJo, LLC).

### Real-time quantitative PCR

Real-time quantitative PCR (RT-qPCR) was performed as we described before [11]. In brief, total RNA from mouse brain cortical tissues or microvessels were isolated at day 1 after TBI with the miRNeasy micro kit (Qiagen, Germantown, MD, USA) according to the manufacturer’s instruction. Complementary DNA (cDNA) was synthesized from 0.5 μg of total RNA using QuantiTect Rev. Transcription Kit (Qiagen). Real-time quantitative PCR was performed using TaqMan® Fast Advanced Master Mix (Applied Biosystems) in a QS3 real-time PCR system (Applied Biosystems). The TaqMan probes used in the study were as follows: Mm00443258_m1 (TNFα), Mm00434228_m1 (IL-1β), Mm01210732_m1 (IL-6), Mm01320970_m1 (VCAM1), Mm00516024_g1 (ICAM1), Mm00441278_m1 (Sele), Mm00441242_m1 (CCL2), Mm01545399_m1 (HPRT). RT-qPCR was carried out in triplicate, and the relative expression of target genes (fold change) was determined using the 2^−ΔΔCt^ method with normalization to HPRT.

### Neutrophil, monocyte, and lymphocyte isolation

To isolate neutrophils, monocytes, and lymphocytes, blood from mice inner canthus were collected in EDTA tubes, followed by centrifuging at 500 g for 15 min (acceleration = 9, no brake). The top plasma layer was carefully removed and the remaining cells were diluted with 4 mL PBS. Next, approximately 5 mL diluted blood was added into 15-mL tubes with the density gradient solution, which was prepared using low-density Histopaque 1077 (Sigma-Aldrich, St. Louis, MO) (5 mL) for the top layer and high-density Histopaque 1119 (4 mL) for the bottom layer. Tubes were then centrifuged at 700 g for 30 min (slow acceleration). Three distinct cell layers were formed, and the uppermost band that contains mononuclear cells (MNCs) and the middle band that contains neutrophil polymorphonuclear cells (PMNs) were collected, separately. Subsequently, 4 volumes of 1% bovine serum albumin (BSA) was added into each tube to dilute the isolated cells and centrifuged at 700 g for another 10 min. Cell pellets were resuspended in 1% BSA and transferred to 1.5-mL tubes. After centrifuging, the isolated MNCs and PMNs were stained with Ly6C-APC (1:100, BioLegend, 128,015) and Ly6G-APC antibodies ((1:100, BioLegend, 127,614), respectively, and further purified using anti-APC magnetic beads (Miltenyi Biotec) according to the manufacturer’s protocol. To isolate lymphocyte, the flow-through-wash fraction of the MNCs was collected, the supernatant was discarded, and the remaining red cells were lysed with BD lysing buffer. The cells were stored at − 80 ℃ before use.

### Membrane protein extraction

Plasma membrane and membrane-associated proteins were extracted with the Mem-PER™ Plus Membrane Protein Extraction Kit following manufacturer’s protocol (89,842, Thermo Scientific). Briefly, after treatment, the neutrophils were harvested by centrifugation at 300 × g for 5 min and washed with cold wash buffer. After washing, neutrophils were added with 0.75 mL of permeabilization buffer plus 100 × protease inhibitor cocktail and vortexed briefly to obtain a homogeneous cell suspension and further incubated for 10 min at 4 ℃ with constant mixing. The permeabilized cells were centrifuged 16,000 × g for 15 min and the pellets were added with 0.5 mL of solubilization buffer and incubated for 30 min at 4 ℃ with constant mixing. After centrifuging at 16,000 × g for 15 min at 4 ℃, the supernatant containing solubilized membrane and membrane-associated proteins were transferred to a new tube and kept at − 80 ℃ for further use.

### Co-immunoprecipitation

Co-immunoprecipitation (Co-IP) was performed as we described previously [21] using Immunoprecipitation kit-dynabeads protein G (Thermofisher, 10007D) according to the manufacturer’s instruction. Briefly, 2 μg of TLR4 antibodies (Santa Cruz, sc-293027), or AXNA2 antibodies (Santa Cruz, sc-28385), or mouse IgG was incubated with protein G-conjugated dynabeads for 15 min at room temperature. The dynabead-antibody complexes were then washed once with antibody binding and wash buffer. After washing, the dynabead-antibody complexes were suspended with 300 μg cell lysates or 100 μg membrane proteins, and incubated for 15 min at room temperature. Subsequently, the precipitates were rinsed with wash buffer three times to remove non-specific binding molecules. The protein was eluted with an elution buffer. The samples were analyzed by Western blotting. All experiments were performed independently for three times.

### Western blotting

Western blotting was performed as we previously described [11]. Proteins of brain samples or cells were prepared with cell lysis buffer (Cell Signaling) with protease inhibitors (Thermo Fisher Scientific) and quantified with the BCA method (Thermo Fisher Scientific). After denaturation, 30 μg of protein samples was separated by 4–12% NuPAGE gel (Thermo Fisher Scientific) and transferred to PVDF membrane, which was further blocked with 5% (w/v) fat-free milk in TBST (tris-buffered saline with 0.1% Tween-20) for 60 min at room temperature. Then, PVDF membrane was incubated overnight at 4 °C with the following primary antibodies: AnxA2 antibody (1:500, Santa Cruz, sc-28385), Ly6G antibody (1:300, BD Pharmingen, 550,291), cleaved-caspase 3 antibody (1:200, Cell Signaling, D175), p65 antibody (1:1000, Cell Signaling, 8242 s), phospho-p65 antibody (1:1000, Cell Signaling, 3033 s), MyD88 (1:500, Santa Cruz, sc-74532), TICAM-1 (TIRF, 1:500, Santa Cruz, sc-514384), TLR4 (1:500, Santa Cruz, sc-293072), Na^+^K^+^ATPase (1:500, Cell Signaling, #3010), and β-actin antibody (1:3000, Sigma, A5441). Subsequently, the PVDF membrane was washed with TBST and further incubated with IRDye 800CW goat anti-Rabbit (1:10,000, LI-COR Biosciences, #926–32,211) or Goat anti-Mouse IgG StarBright Blue 700 (1:10,000, Bio-Rad, #12,004,159) antibodies for 1 h. All images were visualized and captured with Bio-Rad ChemiDocTM MP Imaging System.

### Immunostaining

The mice were sacrificed, and the brain was prepared as described above. Subsequently, the brain was then sliced into 16-µm-thick coronal sections with a Leica Cryostat (Leica, CM1950). Five percent bovine serum albumin (BSA) in PBS with 0.1% Triton X-100 was used to block the brain sections, which were further incubated at 4 °C overnight with the following primary antibodies: Ly-6G antibody (1:100, BD Pharmingen, 551,459), CD45 antibody (1:100, BD Pharmingen, 550,539), cleaved-caspase 3 antibody (1:100, Millipore Sigma, AB3623), neuronal-specific nuclear protein (NeuN) antibody (1:200, Millipore, MAB377). Following washing with TBST, brain sections were incubated with secondary antibody conjugated to fluorescein (1:250, Thermo Fisher Scientific, A32723 and A-11037) for 1 h at room temperature. Sections were washed with PBS and mounted using Vectashield with DAPI. Fluorescence signals in the peri-lesion cortex were observed and captured by a Nikon Ts2R FL microscope (Nikon, Tokyo, Japan). For quantitative analyses, observers were blinded to the experimental groups. The number of positively stained cells per 0.20 square millimeter (0.5 mm × 0.4 mm; 20 × magnification) was counted in three consecutive selected microscopic fields in the peri-lesion cortex as we previously described [11]. The average cell number was calculated for each experimental group.

### Confocal microscopy

Neutrophils were suspended into DMEM/F12 medium plus 1% FBS at 5 × 10^6^/mL and added into 24-well plate with coverslip coated with poly-D-lysine. The cells were stimulated with 2 μg/mL rA2 for 30 min at 37 °C and washed with PBS. Subsequently, cells were fixed in 4% paraformaldehyde for 15 min and were further incubated with rabbit anti-AXNA2 (Abcam, ab41803) and mouse anti-TLR4 (Santa Cruz, sc-293027) overnight at 4 °C. After washing, cells were incubated with Alexa Fluor 594-labeled donkey anti-rabbit IgG and Alexa Fluor 488-labeled goat anti-mouse IgG for 1 h at 4 °C in the dark. Finally, after mounted on glass slides, fluorescence signals on the cells were visualized by using a Leica TCS SP2 confocal microscope at × 600 magnification.

### Cytokine proteome profiler array

The inflammatory cytokines in the mice brain were analyzed using the Mouse Cytokine Proteome Profiler Array Panel A kit (R&D Systems) according to the manufacturer’s specifications. Briefly, 300 μg mice brain samples were incubated for 1 h at room temperature with the supplied cytokine array panel A antibody cocktail. The array membranes were blocked with the blocking buffer, followed by incubating with the lysate-antibody mixtures overnight at 4 ℃ on the platform shaker. After washing in wash buffer, the array membranes were further incubated with streptavidin-HPR in blocking buffer for 30 min at room temperature before mixing with the Chemi reagent mix. Images were then captured with Bio-Rad ChemiDoc™ MP Imaging System. ImageJ was used to quantify and determine spot density.

### Isolation of brain microvessel fragments

Brain microvessels were obtained as we described previously [22]. Briefly, mice were sacrificed at 24 h after TBI and perfused transcardially with ice-cold PBS. Ipsilateral hemispheres were separated, and the cerebellum and white matter were removed. After rinsing with PBS, the ipsilateral cortical tissues were rolled on the filter paper to remove outer meninges using forceps. Then the collected cortical tissues were further homogenized with ice-cold PBS (1 mL for 1 mg of tissue) on ice with Knote Dounce glass tissue grinder (Part 885,300–0002; Kimble Chase Life Science, Vineland, NJ, USA). The homogenate was centrifuged at 4 °C, 2000 × g for 5 min. The supernatant was centrifuged again to increase yield. The two pellets were resuspended with 18% dextran solution (molecular weight 60–90 kDa; USB Corporation, Cleveland, OH, USA) in PBS and then centrifuged again at 4 °C, 2000 g for 20 min. The new pellet was washed in 18% dextran solution and centrifuged at 4 °C, 2000 × g for 15 min. Subsequently, the pellet was further resuspended in 2% BSA, and the suspension was then filtered through a 40-μm cell strainer to get rid of the debris. The microvessels retained on the filter were transferred to microcentrifuge tubes and centrifuged at 4 °C, 12,000 g for 5 min. The resultant microvessel pellet was stored at − 80 °C or directly used for RNA extraction.

### Assessments of motor function and spatial memory

Motor function was assessed on days 1, 3, 7, 14, 21, and 28 after TBI by Rota Rod test as we described before [11, 19]. Briefly, mice were placed on the 4-cm-diameter rotating drum of an accelerating Rota Rod (Harvard Apparatus, Holliston, MA, USA) from 4 to 40 rpm for 5 min, until they were fallen from the rotating drum and the total running time spent on the device was automatically recorded. Each mouse has tested four trials per day with an inter-trial interval of 20 min. The average latency of the four trials was calculated for the analysis. Mice were trained for 2 days, and the baseline was obtained on the day before surgery.

Spatial learning and memory were assessed using a Morris water maze (MWM) test on days 10–14 after TBI, as previously described [18, 19]. Mouse water maze pools (121.92 cm diameter) were filled with water, and a round plastic platform (10.16 cm in diameter and 30.48 in height) in each pool was positioned 1 cm below the surface of the water. Mice were given seven hidden platform trials, including one hidden platform trial that was performed on day 1, and two hidden platform trials were carried out on the following days up to seven hidden platform trials. One probe trial and two visible platform trials were carried out at 24 h after the last hidden trial. For seven hidden platform trials and visible platform trials (with a red flag on the target platform), mice were placed in the tank facing the wall and given 90 s to find and mount the platform. For probe trials, mice were placed in the tanks without the target platform and given 30 s to explore the tank. Ceiling-mounted cameras were used to record the trails, and Any-Maze software (Stoelting Co., Wood Dale, IL) was used to analyze the swim speed, total distance, and time.

Spatial working memory was assessed using a spontaneous alternative Y-maze test as we described before [18, 19]. Mice were placed in the center of the maze and were allowed to explore all three arms for 5 min, which was recorded using a ceiling-mounted camera. The number of arm entries and the number of triads (a set of consecutive arm entries, i.e., triplets of ABC, BCA, CAB) were counted to calculate the alternation percentage. The ratio of correct choice was determined by the following equation: % alternations = [(number of alternations)/(total arm entries − 2)] × 100).

### MAP2 staining and quantitation of traumatic brain tissue loss

MAP2 staining and quantitation of traumatic brain tissue loss were performed as described previously [11]. Five equally spaced brain slices were used: +1.54 to − 3.46 from bregma, with 1000-μm interval. Coronal brain sections were stained with Alexa Fluor® 488-conjugated MAP2 antibody (1:200, MAB3418X, Sigma-Millipore, Burlington, MA, USA) and captured with an image scanner. The brain tissue loss was quantified with ImageJ software and calculated with the following formula: VC − VL (VC is the contralateral hemisphere volume; VL is the volume of residual tissue in the ipsilateral hemisphere).

### Statistical analysis

All data were expressed as mean ± standard error of the mean (SEM) of at least three independently repeated experiments. For measurements such as flow cytometry, mRNA and protein expression levels, immunostaining, and lesion size, statistical analyses between two groups were determined by Student’s *t*-test, and multiple groups were determined by one-way or two-way analysis of variance (ANOVA), followed by the Tukey post hoc multiple comparisons test. For cytokine assays, multiple groups were determined by two-way ANOVA, followed by the test with the original FDR method of Benjamini and Hochberg. Repeated behavioral tests, including Rota Rod, MWM hidden and visible platform tests, were assessed using two-way (treatment × time) repeated measures ANOVA analysis (with trial/time-point as a repeated measures factor) with matched subjects, followed by the Tukey post hoc multiple comparisons test. Single time-point neurobehavioral measurements, including Y-maze and Morris water maze probe tests, were analyzed by one-way ANOVA, followed by the Tukey post hoc multiple comparisons test. All statistical analyses were conducted using GraphPad Prism 7 software (GraphPad Software, Inc., La Jolla, CA). Differences were considered statistically significant at *p* < 0.05.

## Results

### rA2 treatment inhibits peripheral leukocyte activation after TBI in mice

**Fig. 1:**
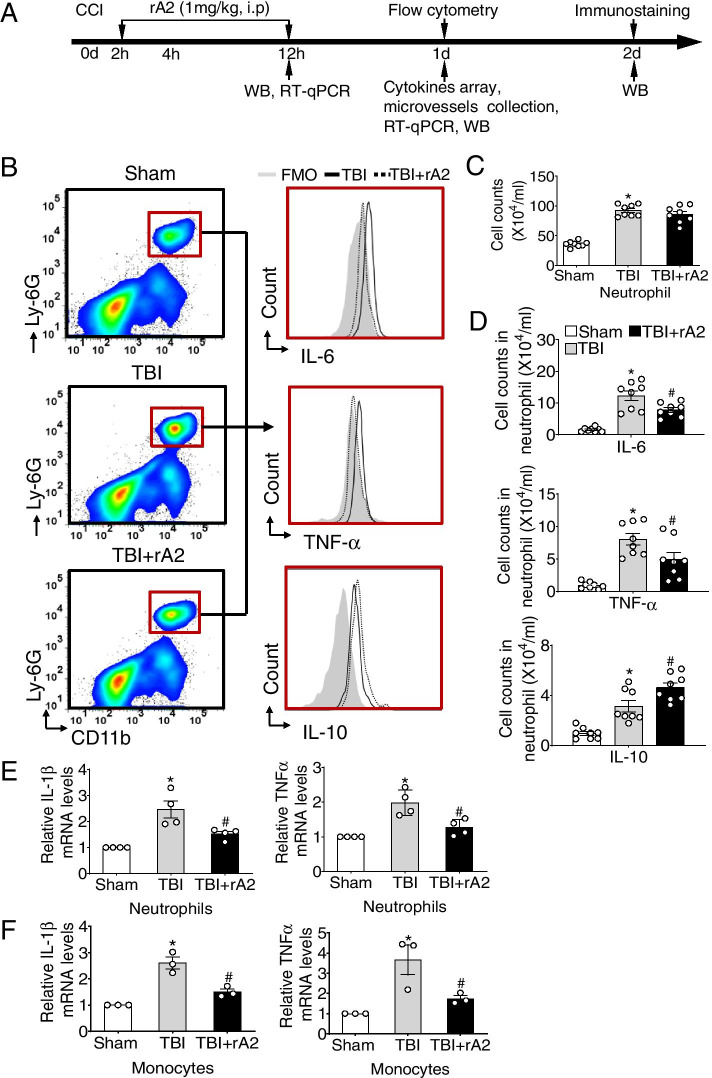
Effect of rA2 on peripheral neutrophils after TBI. **A** Timeline of the experimental procedures for the pathological evaluation. **B** Representative gating strategy of peripheral neutrophils (CD11b^+^Ly6G^+^) and their expression of IL-6, TNFα, and IL-10. All gates were set using FMO controls. **C** Cell counts of peripheral neutrophils of sham, TBI, and TBI + rA2 mice at 24 h after TBI, *n* = 8. **D** Respective bar graphs show the cell counts of IL-6, TNFα, and IL-10 in peripheral neutrophils of sham, TBI, and TBI + rA2 mice at 24 h after TBI, *n* = 8. **E** RT-qPCR analysis of mRNA levels of IL-1β and TNFα in neutrophil that isolated from sham, TBI, and TBI + rA2 mice at 12 h after TBI, *n* = 4. **F** RT-qPCR analysis of mRNA levels of IL-1β and TNFα in monocytes that isolated from sham, TBI, and TBI + rA2 mice at 12 h after TBI, *n* = 3. Data are expressed as mean ± SEM; *p < 0.05 compared to sham; ^#^*p* < 0.05 compared to TBI

The schematic of the experimental protocol is shown in Fig. 1A. We first investigated the effect of rA2 treatment on the count and activation of peripheral circulating leukocytes at 24 h after TBI via flow cytometry analysis. The gating strategy is shown in Fig. 1B and Supplemental Figure 2A. Compared with the sham group, TBI induced a significant increase in the number of neutrophils (Fig. 1C) and monocytes (Supplemental Figure 2B), but not of T lymphocytes (Supplemental Figure 2D). Administration of rA2 had no significant effect on the counts of neutrophils, monocytes, and T lymphocytes, compared with the TBI group (Fig. 1C, Supplemental Figure 2B and D).

We further examined the effect of rA2 treatment in TBI-induced peripheral leukocyte activation at 24 h after TBI. Our results show that rA2 treatment significantly decreased the TBI-induced elevation of pro-inflammatory markers, intracellular IL-6, and TNFα protein, and increased anti-inflammatory marker IL-10 expression in neutrophils (Fig. 1D). Moreover, rA2 also significantly attenuated the TBI-induced TNFα expression in monocytes (Supplemental Figure 2C) and CD69 expression in T lymphocytes (Supplemental Figure 2E). To further determine the effect of rA2 on leukocyte activation induced by TBI, circulating neutrophils and monocytes were isolated at 12 h after TBI, RT-qPCR-quantified mRNA levels of IL-1β and TNFα. Consistent with the flow cytometry data, we detected a significant reduction of IL-1β and TNFα mRNA in the neutrophils (Fig. 1E) and monocytes (Fig. 1F) in the rA2-treated TBI mice compared to the TBI control mice. Taken together, these results indicated that rA2 treatment has a significant inhibitory effect in the circulating neutrophil and monocyte activation after TBI.

### rA2 binds to and reduces TLR4 expression on neutrophil surface

**Fig. 2:**
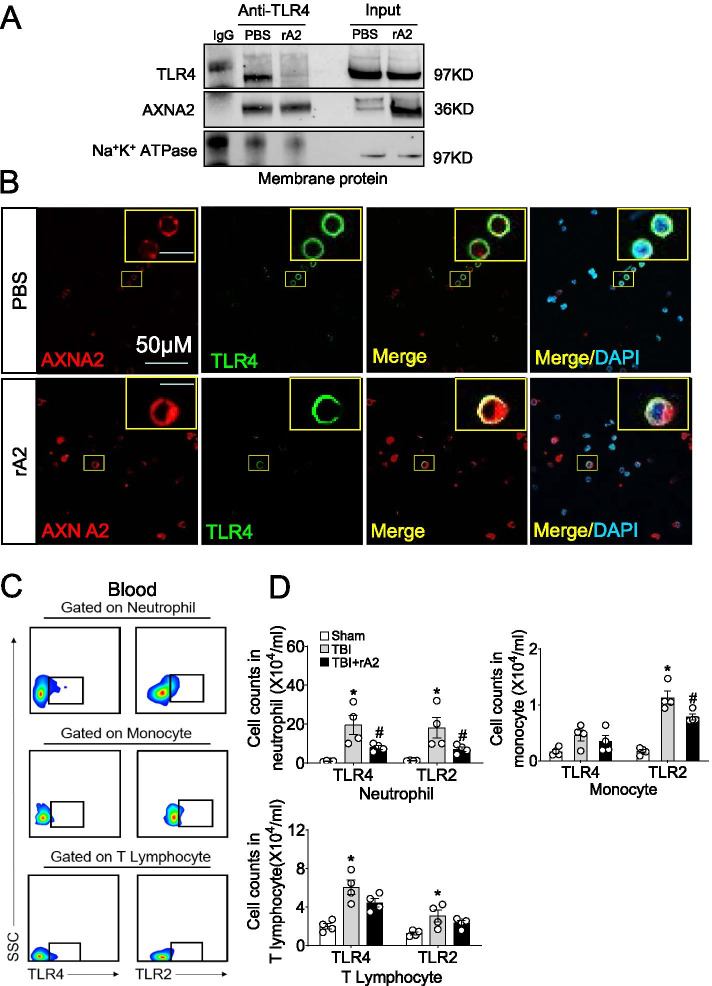
rA2 binds to and reduces TLR4 membrane expression in neutrophils. **A** Representative gel images of Co-IP coupled with Western blotting analysis of the binding of AXNA2 to TLR4 in the membrane of neutrophils, which were treated with PBS or rA2 (2 μg/mL) for 1 h. Anti-TLR4 antibodies were used for immunoprecipitation, and the immunoprecipitates and membrane proteins were analyzed by immunoblotting with anti-AXNA2 antibodies. Na^+^K^+^ATPase served as an equal loading control of cell membrane protein. **B** Representative confocal microscope images of co-immunostained with AXNA2 and TLR4 in neutrophils, which were treated with PBS or rA2 (2 μg/mL) for 1 h. The DAPI was used to stain the nucleus. Scale bar, 50 μm. The scale bar in the yellow box is 10 μm. **C** Representative flow cytometry plots for TLR4^+^ or TLR2^+^ immune cells obtained from blood samples of TBI at 24 h. **D** Quantitation of TLR4 and TLR2 expressing in periphery neutrophils, monocytes, and T lymphocytes from sham, TBI, and TBI + rA2 mice, *n* = 4. Data are expressed as mean ± SEM; *p < 0.05 compared to sham; ^#^*p* < 0.05 compared to TBI

We next explore the potential mechanisms underlying the inhibitory effect of rA2 in pro-inflammatory phenotype of neutrophils and monocytes. It has been reported that TLR4 is increased in the surface of neutrophils from blood or peri-contusional brain tissue, and mediated neutrophil activation in mice after TBI [7], and AXNA2 is capable of interacting with TLR4 and negatively regulating TLR4-triggered inflammatory responses in monocytes [12]. Considering that neutrophils are the most abundant immune cells in circulation and inhibited by rA2, we therefore focus on rA2 binding to TLR4 and the expression alteration of TLR4 on neutrophils surface. rA2 was added into isolated neutrophil for 1 h, and the membrane proteins of the isolated neutrophils were extracted for Co-IP and WB assay. We found that rA2 treatment dramatically reduced TLR4 expression on neutrophil surfaces in vitro via Co-IP assay (Fig. 2A). Moreover, we also found that an almost equal amount of AXNA2 can be immunoprecipitated by TLR4 antibody in PBS and rA2 treatment groups, although there were less TLR4 proteins in the membrane of rA2-treated neutrophils compared with PBS-treated neutrophils (Fig. 2A), indicating that rA2 treatment increases the interaction of AXNA2 with TLR4 on the membrane of neutrophils. Moreover, confocal microscope showed that rA2 treatment increases the co-localization of AXNA2 with TLR4 and dramatically reduces TLR4 localization on the neutrophil surface (Fig. 2B). To further investigate whether rA2 administration reduces TLR4 expression on the neutrophil surface in vivo, flow cytometry was performed (Fig. 2C). We detected a marked increase of TLR4 and TLR2 surface protein expression on circulating and brain-infiltrating neutrophils, monocytes, and T lymphocytes at 24 h after TBI (Fig. 2D, Supplemental Figure 3C-D). However, rA2 treatment significantly inhibited the increase of TLR4 and TLR2 membrane expression in circulating neutrophils, and rA2 treatment also reduced TLR2 membrane expression in neutrophils but had no inhibitory effect on TLR4 membrane expression in monocytes and T lymphocytes (Fig. 2D). Moreover, rA2 treatment has inhibitory tendency, but did not significantly alter the expression of the TLR4 and TLR2 in brain-infiltrated leukocytes after TBI (Supplemental Figure 3D). These in vitro and in vivo data suggest that rA2 binds to and reduces TLR4 on neutrophil surfaces.

### rA2 treatment attenuates TLR4-NFκB signaling in neutrophils after TBI in mice

**Fig. 3:**
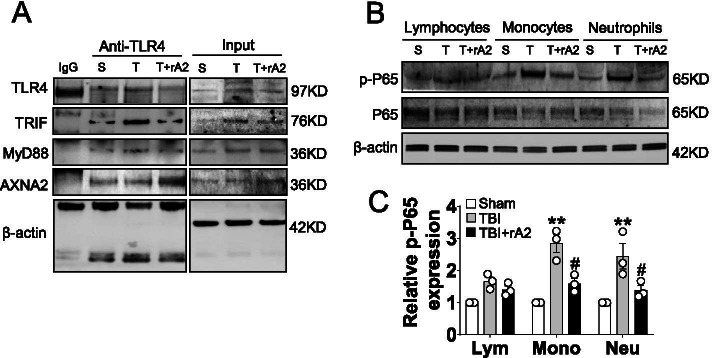
Effect of rA2 on TLR4/NFκB activation in leukocytes after TBI. **A** Representative gel images of Co-IP coupled with Western blotting analysis of the binding of TLR4 to TRIF, MyD88, and AXNA2 in isolated neutrophils of sham (S), TBI (T), and TBI + rA2 (T + rA2) mice at 12 h after TBI. **B** Representative gel images of Western blotting for p-P65, P65, and β-actin expression in isolated lymphocytes (Lym), monocytes (Mono), and neutrophils (Neu) of sham (S), TBI (T), and TBI + rA2 (T + rA2) mice at 12 h after TBI. **C** Quantitative analysis of p-P65 expression, *n* = 3. Data are expressed as mean ± SEM; **p < 0.01 compared to sham; ^#^*p* < 0.05 compared to TBI

To ask whether rA2 could inhibit TLR4-mediated pro-inflammatory activation of neutrophils after TBI, the Co-IP approach combined with Western blotting analysis was used to determine the interaction of TLR4 with its downstream signaling molecules, including TRIF and MyD88. Our results showed that there was increased binding of TLR4 to TRIF and MyD88 in isolated circulating neutrophils at 12 h after TBI, and the increased binding was reduced by rA2 treatment (Fig. 3A). These results show that rA2 attenuates pro-inflammatory TLR4 signaling activation in neutrophils after TBI.

TLR4 signaling through the adaptor molecule MyD88 and TRIF leads to the activation of transcriptional factor NFκB, a pro-inflammatory gatekeeper, and subsequent cytokine production [23, 24]. Next, we ask whether rA2 inhibits NFκB activation. At 12 h after TBI, we detected a significant increase in the phosphorylated p65 (p-p65), the common activation marker of NFκB, in the circulating monocytes and neutrophils. Increased p-p65 levels were significantly attenuated in the TBI + rA2 group (Fig. 3B, C). Taken together, these data indicate that rA2-inhibited TLR4-NFκB signaling cascade is involved in the inhibitory effect on pro-inflammatory neutrophils at the early phase of TBI.

### rA2 treatment attenuates neutrophil infiltration and activation in the mouse brain after TBI in mice

**Fig. 4:**
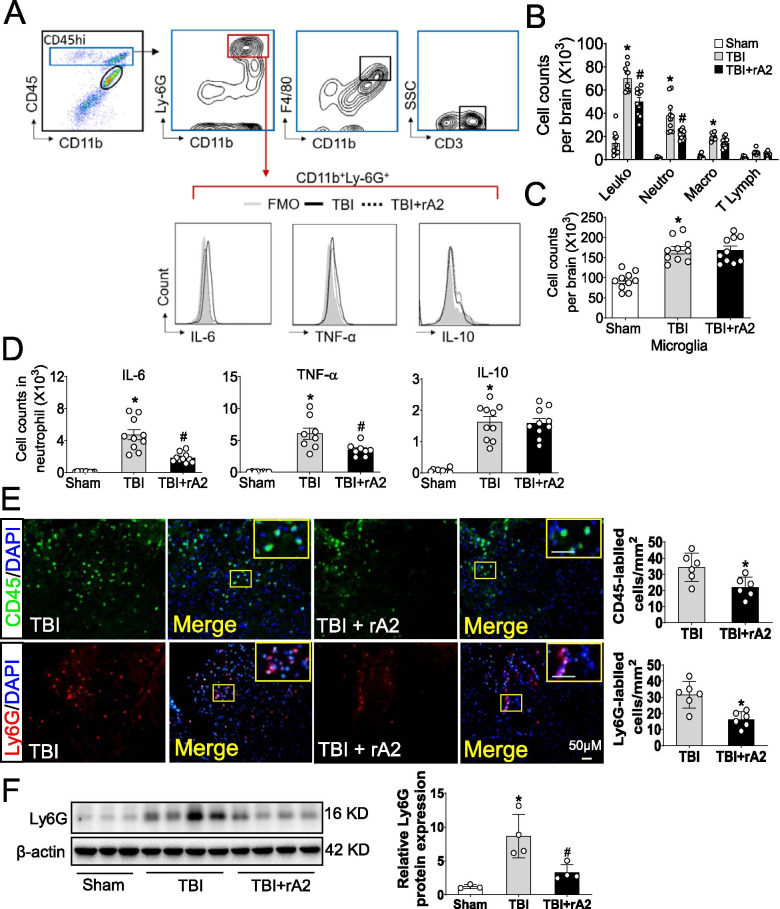
Effect of rA2 on TBI-induced neutrophil infiltration and activation in the mouse brain. **A** Single-cell suspensions were prepared from brain tissues of mice with TBI at 24 h after surgery. Flow cytometry plots show gating strategy of brain-infiltrated immune cells including microglia (CD11b^+^CD45^int^), neutrophils (CD11b^+^CD45^high^Ly6G^+^), monocyte/macrophages (CD11b^+^CD45^high^F4/80^+^), and T lymphocytes (CD45^high^CD3^+^), and the expression of IL (interleukin)-6, TNFα (tumor necrosis factor-α), and IL-10 in neutrophils. All gates were set using FMO controls. **B** Quantitative analysis of CNS-invading leukocytes in the brains of sham, TBI, and TBI + rA2 mice, *n* = 10. **C** Quantitative analysis of microglia in the brains of sham, TBI, and TBI + rA2 mice, *n* = 10. **D** Quantification of neutrophil expressing IL-6, TNFα, and IL-10 in the brains of sham, TBI, and TBI + rA2 mice, *n* = 8–10. **E** Representative immunostaining images and quantification of CD45- and Ly6G-positive cells in the cortex area of the ipsilateral hemisphere in sham, TBI + saline, and TBI + rA2 mice brain. Scale bar, 50 μm. Data are expressed as mean ± SEM; *n* = 6, *p < 0.05 compared to TBI. **F** Representative gel images of Western blotting and quantitative analysis for Ly-6G expression in the cortex area of the ipsilateral hemisphere in C57BL6 mice at 2 days after TBI, *n* = 3–4. Data are expressed as mean ± SEM; *p < 0.05 compared to sham; ^#^*p* < 0.05 compared to TBI

In the next experiment, we performed flow cytometry to investigate the effect of rA2 treatment on leukocyte brain infiltration at 24 h after TBI. The gating strategy of immune cell subsets is shown in Fig. 4A and Supplemental Figure 3A. The results showed that there was a significant increase in the total numbers of leukocytes (CD45^high^), neutrophils (CD11b^+^CD45^high^Ly6G^+^), macrophages (CD11b^+^CD45^high^F4/80^+^), and microglia (CD11b^+^CD45^int^) in the TBI brains (Fig. 4B, C). Notably, neutrophils comprised the majority of infiltrating leukocytes (54.4%) at 24 h post-TBI. We find that administration of rA2 significantly lowered infiltrated leukocytes (20.1% reduction) and neutrophils (16.4% reduction) in the brain at 24 h post-TBI when compared with the TBI vehicle group (Fig. 4B). In contrast, rA2 did not significantly affect the number of macrophage and T lymphocytes, and microglia in the TBI mouse brains (Fig. 4B, C). Consistently, our immunostaining data showed that rA2 treatment significantly reduced invasion of CD45- (a marker for leukocytes) and Ly6G (a marker for neutrophil)-labeled cells in the cortical peri-lesional area of the ipsilateral brain at 24 h post-TBI (Fig. 4E). Western blotting analysis further verified that Ly6G protein levels were significantly reduced in rA2-treated TBI brain samples at 48 h post-TBI, compared to the saline-treated TBI samples (Fig. 4F). Next, we examined the effect of rA2 treatment on the activation of brain-infiltrated neutrophils, macrophages, lymphocytes, and resident microglia. We found that rA2 treatment significantly lowered IL-6 and TNFα expression in brain-infiltrating neutrophils and CD69 in brain-infiltrated T lymphocytes at 24 h after TBI (Fig. 4D, Supplemental Figure 3B). In contrast, rA2 did not alter expression of IL-10 in the brain-infiltrating neutrophils, CD86, IL-6, and TNFα in microglia and macrophage and did not alter IL-6 and TNFα in T lymphocytes at 24 h after TBI (Fig. 4D, Supplemental Figure 3B), indicating rA2 treatment mainly attenuates neutrophil activation in the mouse brain after TBI in mice. Collectively, these data suggest that rA2 inhibits neutrophil infiltration and activation in the mouse brain after TBI in mice.

### rA2 inhibits pro-inflammation in the brain after TBI in mice

**Fig. 5:**
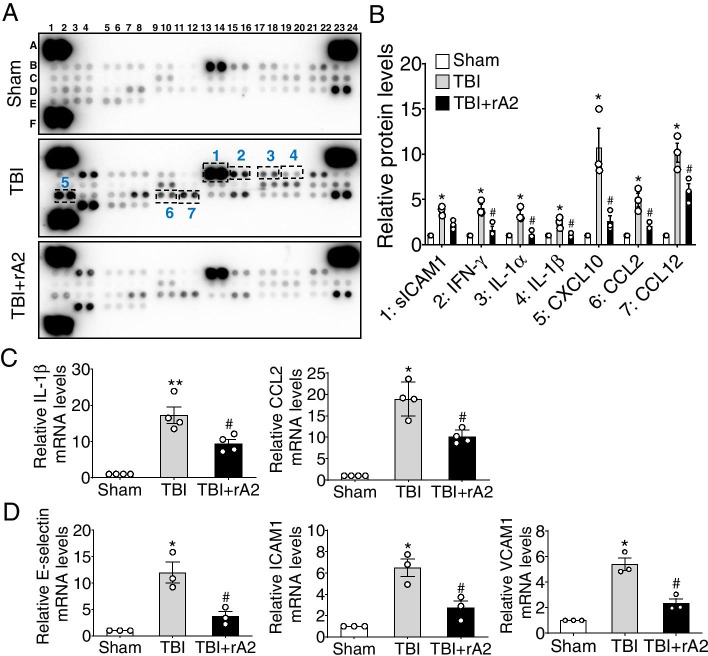
Effect of rA2 treatment on TBI-induced neuroinflammation. **A** Representative cytokine profile array in the peri-lesion cortex of the ipsilateral hemisphere in sham, TBI, and TBI + rA2 mice at 1 day after TBI. **B** Quantitation of cytokines, *n* = 3. **C** Quantitative analysis of IL-1β and CCL2 mRNA levels in the cortex area of the ipsilateral cortex in sham, TBI + saline, and TBI + rA2 mice at 12 h after TBI, *n* = 4. **D** Quantitative analysis of E-selectin, VCAM1, and ICAM1 mRNA levels in the isolated cortical microvessels at 24 h after TBI, *n* = 3. Data are expressed as mean ± SEM; *p < 0.05 compared to sham; ^#^*p* < 0.05 compared to TBI

Brain-infiltrating neutrophils aggravate neuroinflammation and brain damage during the acute phase of TBI [25]. Next, we aimed to test the effects of rA2 administration in the neuroinflammation after TBI. We first asked whether rA2 administration could alter the neuroinflammatory profiles of brain tissues after TBI. A commercial cytokine protein array kit was used to examine the expression profile of inflammatory cytokines and chemokines. Our results showed that TBI dramatically increased the protein expression of IFN-γ, IL-1α, IL-1β, CXCL10, CCL2, and CCL12 in the ipsilateral cortex at 24 h after TBI, and expression levels were significantly attenuated by rA2 administration (Fig. 5A, B). RT-qPCR was used to validate the mRNA levels of selected cytokines, IL-1β and CCL2, in the ipsilateral cortex at 12 h after TBI. The mRNA data were consistent with the protein array results (Fig. 5C). We also examined the effects of rA2 administration on the expression of cerebrovascular inflammation markers as well as endothelial adhesion molecules in the isolated ipsilateral microvessels at 24 h after TBI. Our results exhibit that rA2 administration significantly attenuated TBI-induced mRNA expression elevation of ICAM1, VCAM1, and E-selectin (Fig. 5D), indicating an inhibitory effect of rA2 in TBI-induced cerebrovascular inflammation. In all, these experimental data suggest that rA2 treatment attenuates TBI-induced pro-inflammatory responses in the brain.

### rA2 treatment reduces neurodegeneration after TBI in mice

**Fig. 6:**
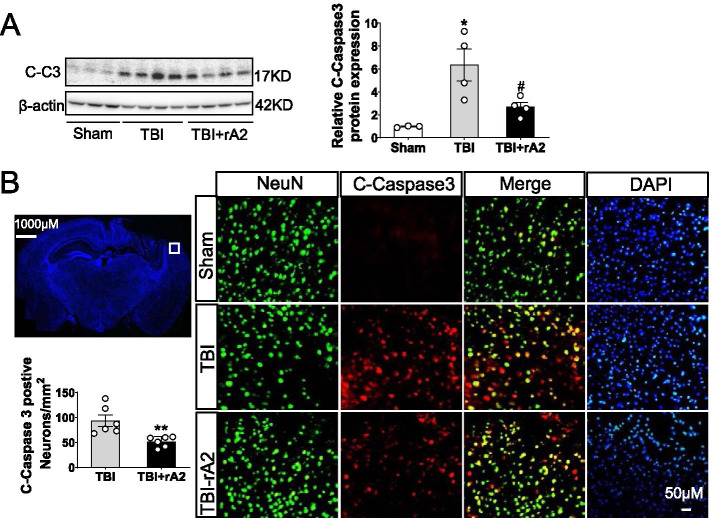
Effect of rA2 on neurodegeneration after TBI. **A** Representative gel images and quantification of Western blotting for cleaved-caspase 3 on day 2 after TBI, *n* = 3–4. Data are expressed as mean ± SEM; *p < 0.05 compared to sham; ^#^*p* < 0.05 compared to TBI. **B** Representative cleaved-caspase 3 and NeuN double-staining images and quantitation of cleaved-caspase 3-positive neurons. Scale bar, 50 μm. The white box in the representative immunostaining indicates the image field of cleaved-caspase 3 and NeuN double staining in the ipsilateral cortex, *n* = 6. Data are expressed as mean ± SEM; **p < 0.01 compared to TBI

Early pro-inflammatory activation of neutrophil is positively associated with increased secondary neurodegeneration and worsened neurological outcome after TBI [5]. We next analyzed the effect of rA2 treatment on neurodegeneration after TBI. Western blot analysis showed that the neurodegenerative biomarker cleaved-caspase 3 (C-caspase 3) level was significantly increased in the ipsilateral cortex at 48 h post-TBI, but this increase was significantly inhibited by rA2 administration (Fig. 6A). Double immunostaining of the neuronal-specific nuclear protein (NeuN) and C-caspase 3 showed most active caspase 3 signals were co-localized with NeuN-positive neurons at the peri-lesion cortex at 48 h after TBI, but the double-positive cell numbers were significantly reduced by the rA2 administration (Fig. 6B). These results suggested that rA2 treatment significantly reduces neurodegeneration after TBI in mice.

### rA2 treatment improves long-term neurological outcomes and reduces brain tissue loss after TBI in mice

**Fig. 7:**
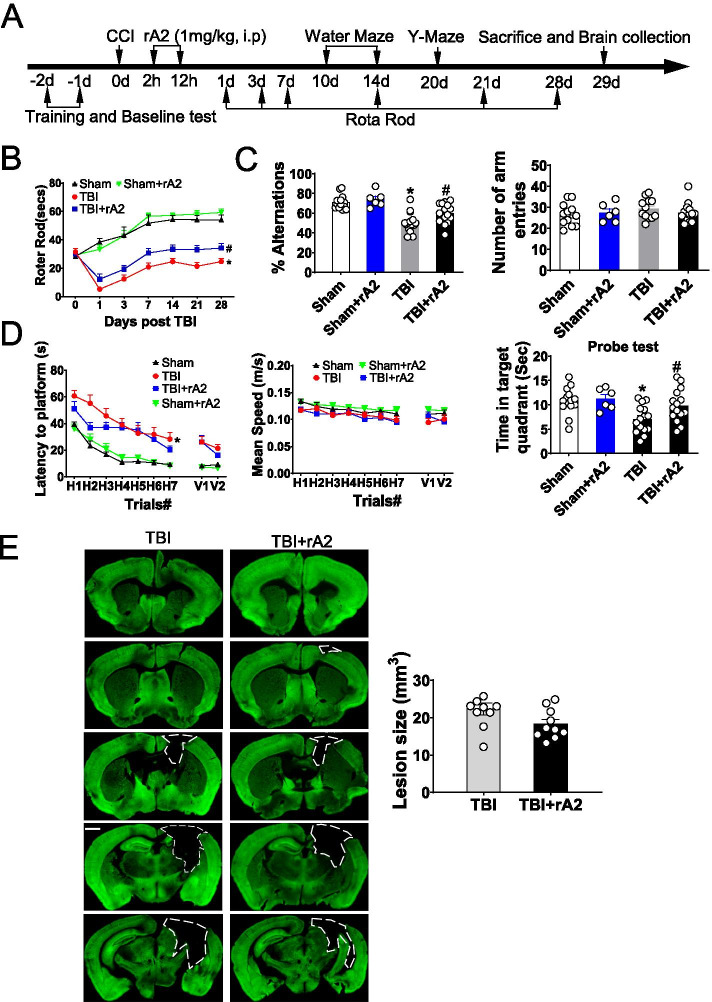
Effect of rA2 on the neurobehavioral outcome and brain tissue loss after TBI. **A** Schematic diagram showing the timeline of the experimental procedures for neurobehavioral assessment. **B** Rotor-Rod test. **C** Y-maze. **D** Morris water maze was performed to assess neurobehavioral outcomes up to 28 days after TBI, *n* = 8–15. Data are mean ± SEM; *p < 0.05 compared to sham; ^#^*p* < 0.05 compared to TBI. **E** Representative MAP2 staining and quantification of brain tissue loss. The dashed line indicates the border of the brain tissue loss. Scar bar, 1 mm. *n* = 10 per group. Data are mean ± SEM; *p < 0.05 compared to TBI

Next, a battery of neurobehavioral tests was used to evaluate the effects of rA2 administration in neurological deficits after TBI (Fig. 7A). For rA2 administration after TBI, the Rota Rod test for sensorimotor function showed a significant improvement in the functional deficit compared to the vehicle group (Fig. 7B). Y-maze test data showed that rA2 administration significantly increased the alternation ratio without altering the total number of arm entries (Fig. 7C). Moreover, Morris water maze results exhibited significantly worse learning and memory ability in TBI mice versus sham control (Fig. 7D). rA2 administration had no significant effect in the time spent to find the platform, swimming speed, and distance in the hidden trials compared to vehicle control mice after TBI; however, rA2-treated TBI mice presented a significant preference for the target quadrant in the probe test (Fig. 7D). These neurobehavioral test results suggest that rA2 administration improves sensorimotor function and working memory after TBI.

Lastly, brain tissue loss was quantified at 29 days after TBI via MAP2 staining. Our results showed rA2 had no significant effect on TBI-induced brain tissue loss (Fig. 7E). Taken together, these results suggest that rA2 administration at the acute phase after TBI may inhibit pro-inflammatory profiles in brain tissue, therefore consequently reduce per-lesion neurodegeneration and neurological deficits after TBI.

## Discussion

**Fig. 8:**
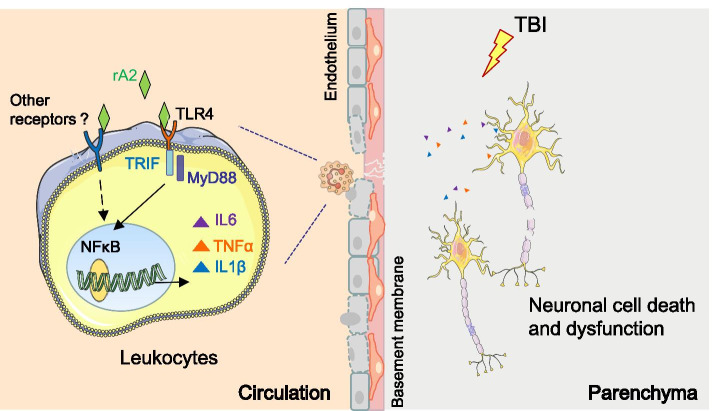
Proposed mechanisms of acute treatment of rA2 in protecting against TBI. rA2 binds to and reduces TLR4 on leukocyte surface, and attenuates TLR4/NFκB activation in leukocytes, and thereby inhibits leukocyte brain infiltration, neuroinflammation, and neuronal cell death, and ultimately improves neurological outcome after TBI

In the present study, we investigated the mechanism and therapeutic effect of rA2 administration after TBI in mice. Our major experimental findings are summarized in the following: (1) rA2 administration at 2 h after TBI significantly reduced pro-inflammatory activation of hematogenous leukocytes, particularly neutrophils and monocytes after TBI; (2) in neutrophils, rA2 binds to and reduces TLR4 on neutrophil surface and disrupts the pro-inflammatory TLR4-NFκB signaling; (3) rA2 administration mainly attenuates neutrophil infiltration and activation in the mouse brain in the acute phase after TBI; (4) rA2 also inhibits neuroinflammation after TBI; (5) rA2 reduces neurodegeneration after TBI; (6) rA2 improves long-term neurological outcomes and reduces brain tissue loss after TBI. The experimental results of present study support our hypothesis, indicating a therapeutic potential of rA2 administration in protecting against secondary injury of TBI by suppressing pro-inflammatory activation of circulating leukocytes and their brain infiltration, particularly neutrophils. The working model of rA2 administration after TBI is proposed in Fig. 8.

Neutrophils are the most abundant leukocytes and the major cell population of rapidly recruited peripheral leukocytes to the injured brain, where they amplify brain damage by aggravating neuroinflammation during the acute phase of brain injury [3, 4]. Consistently, our data revealed that neutrophils comprise the majority of the brain-infiltrating leukocytes (54.4%) at 24 h after severe TBI, whereas macrophages and T lymphocytes only account for 28.3% and 9.2%, respectively. Therefore, targeting the activation and infiltration of neutrophils in the acute phase of TBI is crucial for preventing neuroinflammation-associated neurodegeneration [1]. Depletion of neutrophils with anti-Gr-1 antibodies following TBI is reported to reduce apoptosis, edema, microglia/macrophage activation, and lesion size [26]. Here, we demonstrate that rA2 treatment significantly inhibited TBI-induced neutrophil activation and brain infiltration of neutrophils at 24 h after TBI. However, as rA2 treatment did not affect infiltrating macrophage and T lymphocytes at 24 h after TBI, this strongly suggests that the beneficial effect of rA2 treatment in the acute phase is primarily reliant on its molecular interaction with neutrophils after TBI. In agreement with our observations, rA2 treatment has been reported to reduce neutrophil infiltration 24 h, but reduced extravasated macrophages at 7 and 42 days in injured spinal cord after contusion [16]. Therefore, rA2 mainly attenuates neutrophil activation and infiltration at the early acute stage of TBI.

In this study, we discover that rA2 binds to neutrophilic TLR4, leading to the reduction of TLR4 expression and attenuates the TLR4/NFκB signaling in response to TBI. TLR4 expression is low under physiological resting conditions, but it may be increased and translocated to the cell membrane upon pathological stimulation [27]. It is clearly recognized in multiple brain injury pathologies that TLR4 activation plays a crucial role in the pro-inflammatory activation of leukocytes, including TBI [7] and cerebrovascular disorders [28]. One study reports that endogenous AXNA2 can control TLR4 internalization and negatively regulates TLR4-triggered inflammatory responses in murine macrophage cell lines [12]. Recently, Vaibhav et al. find that TLR4 expression on the neutrophil membranes are significantly increased after TBI and associated with poor outcome [7]. There are two TLR4 activation-mediated pathways, including the MyD88-dependent pathway, which is associated with NFκB activation and cytokine production [23], as well as the TRIF-dependent pathway, which generates type I interferon such as IFNα/β through interferon regulatory factor (IRF-3) and via activation of late-phase NFκB [24]. Our results suggest that rA2 administration simultaneously blocks both TLR4/MyD88 and TLR4/TRIF signaling pathways following TBI. The underlying mechanisms for the inhibitory effect of rA2 on both TLR4/MyD88 and TLR4/TRIF signaling pathways in neutrophils may be complicated, but probably due at least in part to the properties of rA2 as an antagonist of TLR4. Interestingly, our flow cytometry results show that rA2 treatment can also reduce neutrophilic TLR2 expression after TBI. It has been reported that AXNA2 interacts with TLR2 [29]. These studies indicated that TLR2 may also partially mediate rA2-inhibited neutrophil activation. However, it has been found the A2t, which consisted of two AXNA2 and two S100A10 (p11), modulates macrophage function through TLR4, but not TLR2 [14]. Moreover, S100A10, the binding partner of AXNA2, has also been reported to bind to TLR4 and inhibit TLR signaling [30]. Therefore, based on ours and other’s findings, we believe that the inhibitory effects of rA2 on neutrophil are mainly mediated by TLR4. However, we still cannot rule out the possibility that TLR2 or other receptors may also be involved in rA2-mediated inhibition of neutrophil activation after traumatic brain injury in this study, which needed to be further elucidated in our future study.

It is noteworthy that there may be distinct roles of endogenous AXNA2 versus exogenous rA2. For example, endogenous AXNA2 interacts with and acts as a co-receptor of TLR4, mediates anti-β2-glycoprotein I/β2-glycoprotein I (anti-β2 GPI/β2 GPI)-induced TLR4/MyD88 and TLR4/TRIF signaling pathways [13], indicating a crucial role for the membrane AXNA2-TLR4 co-receptors in mediating macrophage activation, which is seemly contrary to our experimental findings of rA2 effects. Previous studies report that addition of purified two soluble domains of AXNA2, including domains I and IV, could act as a dominant-negative competitor of extracellular AXNA2 by competing for ligands, blocking the ensuing cellular response [31]. Blocking of membrane AXNA2 with antagonist, TM601, mitigated NFκB activation in epithelial cells [32]. We therefore speculate that rA2 may be capable of binding to the extracellular domain of membrane AXNA2 and co-receptor TLR4, and block membrane TLR4/AXNA2-associated cellular activation and signaling. Interestingly, similar phenotypes were reported in other pro-inflammatory receptors, as soluble TLR2 acts as a decoy receptor by associating with its co-receptor CD14, thus negatively regulating TLR2-mediated inflammatory responses by disrupting the interaction [33]. In addition, soluble RAGE also exerts a decoy function and inhibits RAGE signaling through competition with membrane-bound RAGE for ligand binding [34]. Therefore, we speculate that rA2 could be a decoy co-receptor of TLR4 and inhibits TLR4/AXNA2 co-receptor-mediated signaling pathways. Although the role of the endogenous membrane AXNA2 is out of the scope of the present study, it is important to elucidate the role and molecular mechanisms of endogenous AXNA2 and interaction with rA2 in modulating leukocyte activation after brain injury.

TBI can cause excitotoxic cell death at the first hours in the local lesion site, and apoptotic cell death later, particularly at the lesion site’s surrounding cortex area after primary injury [35]. TBI also leads to hippocampal damage, which was characterized by progressive neuronal death and hippocampal atrophy [36]. In this study, neurodegeneration in the hippocampus area had not been determined due to the significant loss of hippocampus structure in our severe CCI model. Considering under most conditions, only small molecules (molecular weight < 600 Da, chain length < 6 amino acid) and lipid soluble molecules could across the blood–brain barrier (BBB) and then enter the brain [37], it is unlikely that rA2 treatment can penetrate BBB and directly protect brain cells. It is known that peripheral leukocytes contribute to the secondary brain damage in TBI by mediating detrimental pro-inflammatory reaction in the brain [5]. Neutrophils are reported to home to the injured sites within hours after TBI [2] and have specifically been identified as one of the critical pathological factors causing neurodegeneration and neurological deficits after TBI [5, 26]. Thus, inhibition of brain neutrophil infiltration and neuroinflammation by rA2 might be an important reason to reduce neuronal cell death. As expected, our experimental results demonstrate that rA2 inhibits TBI-induced pro-inflammatory responses in the brain at 24 h post-TBI and inhibits TBI-induced apoptotic caspase 3 activations in peri-lesional cortical neurons at 48 h post-TBI. Consistently, we demonstrate that rA2 administration significantly improved the sensorimotor function and working memory after TBI, although rA2 administration did not significantly improve the overall learning and memory in the Morris water maze test. Moreover, rA2 showed a reduced tendency, but did not significantly reduce lesion volume at 29 days after TBI. These experimental results indicate rA2 might be developed as an effective therapeutic approach against neuroinflammation-associated secondary brain damage after TBI. In light of these results, the optimization of rA2 therapeutic regimen and other translational aspects should be further investigated.

There are several limitations in this study. First, although the half-life of ANXA2 has been determined, the pharmacokinetics of rA2 still needed to be fully investigated in the future. Understanding the pharmacokinetic properties of rA2, including distribution, absorption, metabolism, and excretion, will be of great value for the preclinical evaluation on the pharmacological function and translational potential of rA2. Second, our data showed that rA2 also inhibited pro-inflammatory cytokine TNFα and IL-1β, p-P65, and TLR2 expression in circulating monocytes at 24 h after TBI, implying that the inhibitory effect of rA2 on neuroinflammation is partially mediated by modulating inflammatory phenotype of circulating monocytes. Moreover, although the inhibitory effect of rA2 on neutrophils seems to be dominant at 24 h after TBI, monocytes/macrophages may outnumber the infiltrated neutrophils and become the main targets of rA2 treatment at the delayed phase of TBI. Therefore, the role of rA2 in modulating monocyte/macrophage-associated neuroinflammation after TBI needs to be further investigated. Third, our experimental results suggest rA2 attenuation of post-TBI pro-inflammatory activation of neutrophils, consequently reducing brain infiltration and associated detrimental neuroinflammation. However, the reduction of neutrophilic infiltration can also be attributed to BBB integrity protection by the rA2 administration, which needs to be carefully dissected in the future. Fourth, there are likely other TLR4-NFκB-independent signaling modulations of rA2 administration; for example, our group has demonstrated that rA2 can also affect cerebrovascular function [38] and coagulation/fibrinolysis [21], all of which warrants further investigation in the TBI animal models. Fifth, although we show that rA2 might be effective against neuroinflammation-associated neurodegeneration after TBI, further investigations are needed to optimize the rA2 administration regimen and evaluate all translational aspects in preclinical animal models.

## Conclusions

In summary, our present study demonstrates that rA2 inhibits peripheral leukocyte activation and brain infiltration after TBI. The underlying molecular mechanism might be at least in part attributed to rA2 bindings to pro-inflammatory receptor TLR4 and blocking TLR/NFκB signaling activation pathways. Taken together, although the therapeutic roles and molecular mechanisms need to be further defined and deeply elucidated, the present study demonstrates rA2 administration is beneficial for improving neurological outcomes after TBI at least in part by suppressing neuroinflammation-associated neurodegeneration, suggesting rA2 might be developed as a new and effective approach for the treatment of TBI.

## Supplementary Information

**Additional file 1: Supplemental Figure 1.** Plasma concentration of Annexin A2. The plasma concentrations of Annexin A2 in C57BL/6 mice after single i.p. injection of rA2 (1 mg/kg) were measured using ELISA at 0 h, 2 h, 6 h, 12 h, 24 h, 48 h, and 72 h post-injection (i.p.). The concentrations are expressed as micrograms of Annexin A2 per milligram of the plasma. *n* = 3 mice for each time point. Data are expressed as mean ± SEM.

**Additional file 2: Supplemental Figure 2.** Effect of rA2 on monocytes and T lymphocytes in blood after TBI. (A) Representative gating strategy of peripheral monocytes (CD11b^+^ Ly6C^+^), T lymphocytes (CD3^+^) from single-cell suspensions, and the expression of IL-6, TNF-α, IL-10 in monocytes, and the expression of IL-6, TNF-α, CD69 in T lymphocytes. All gates were set using FMO controls. (B,D) Counts of peripheral monocytes (B) and T lymphocytes (D) of Sham, TBI, and TBI + rA2 mice at 24 h after TBI, *n* = 6–8. (C) Quantitative analysis shows the expression of IL-6, TNF-α, and IL-10 in monocytes, *n* = 4–6. (E) Quantitative analysis shows the expression of IL-6, TNF-α, and CD69 in T lymphocytes, *n* = 4–6. Data are expressed as mean ± SEM, * *p* < 0.05 compared to sham, # *p* < 0.05 compared to TBI.

**Additional file 3: Supplemental Figure 3.** Effect of rA2 on the activation of microglia, macrophage and T lymphocytes in the mouse brain. (A) Representative gating strategy for the expression of CD86, IL-6, TNF-α in microglia (CD11b^+^CD45^int^) and macrophages (CD11b^+^ CD45^high^ F4/80^+^), and the expression of CD69, IL-6, TNF-α in T lymphocytes (CD45^+^ CD3^+^), the brain tissues obtained at 24 h after TBI from Sham, TBI, and TBI + rA2 mice. All gates were set using FMO controls. (B) Cell counts of microglia expressing CD86, IL-6, and TNF-α in the brain, and cell counts of macrophage expressing CD86, IL-6, and TNF-α in the brain, and cell counts of T lymphocyte expressing CD69, IL-6, and TNF-α in brains, *n* = 4–8. (C) Flow cytometry plots show the expression of TLR4 and TLR2 in brain infiltrated neutrophils, macrophages, and T lymphocytes obtained from brain tissues of TBI at 24 h. (D) Quantitation of TLR4 and TLR2 expressing in brain infiltrated neutrophils, macrophages, and T lymphocytes of Sham, TBI, and TBI + rA2 mice, *n* = 4. Data are expressed as mean ± SEM, * *p* < 0.05 compared to Sham, # *p* < 0.05 compared to TBI.

## Data Availability

All data generated or analyzed during this study are included in this published article.

## Abbreviations

TBI: Traumatic brain injury
CCI: Controlled cortical impact
TBST: Tris-buffered saline with 0.1% Tween-20
rA2: Recombinant annexin A2
AXNA2: Annexin A2
BBB: Blood–brain barrier
WB: Western blotting
RT-qPCR: Real-time quantitative polymerase chain reaction
IL-1β: Interleukin-1 beta
TNFα: Tumor necrosis factor-α
NFκB: Nuclear factor kappa B
TLR4: Toll-like receptor 4
FMO: Fluorescence minus one
ANOVA: One-way analysis of variance
