# Supplementary figures and images for: Recombinant annexin A2 inhibits peripheral leukocyte activation and brain infiltration after traumatic brain injury
Source: J Neuroinflammation. 2021 Aug 9;18:173. doi: 10.1186/s12974-021-02219-7 (PMC8353736; doi:10.1186/s12974-021-02219-7)

# Supplemental Figure 1

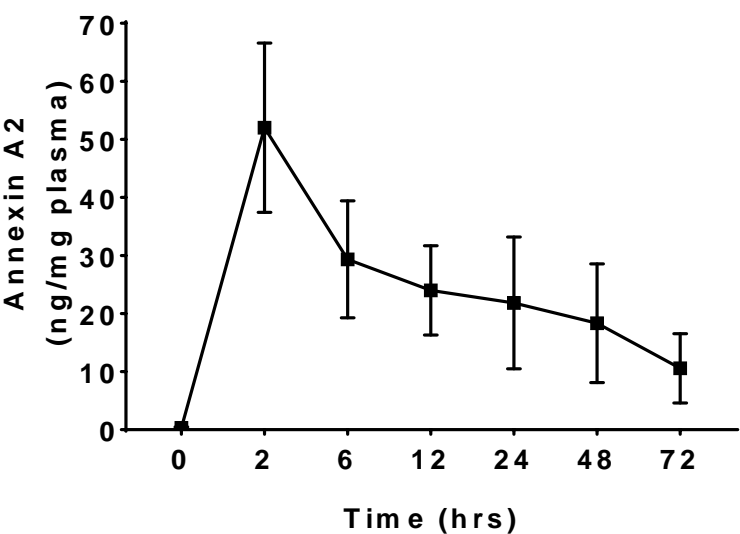

Supplement: Supplementary file 1 — Additional file 1: Supplemental Figure 1. Plasma concentration of Annexin A2. The plasma concentrations of Annexin A2 in C57BL/6 mice after single i.p. injection of rA2 (1 mg/kg) were measured using ELISA at 0 h, 2 h, 6 h, 12 h, 24 h, 48 h, and 72 h post-injection (i.p.). The concentrations are expressed as micrograms of Annexin A2 per milligram of the plasma. n = 3 mice for each time point. Data are expressed as mean ± SEM. [file 12974_2021_2219_MOESM1_ESM.pdf]

# Supplemental Figure 2

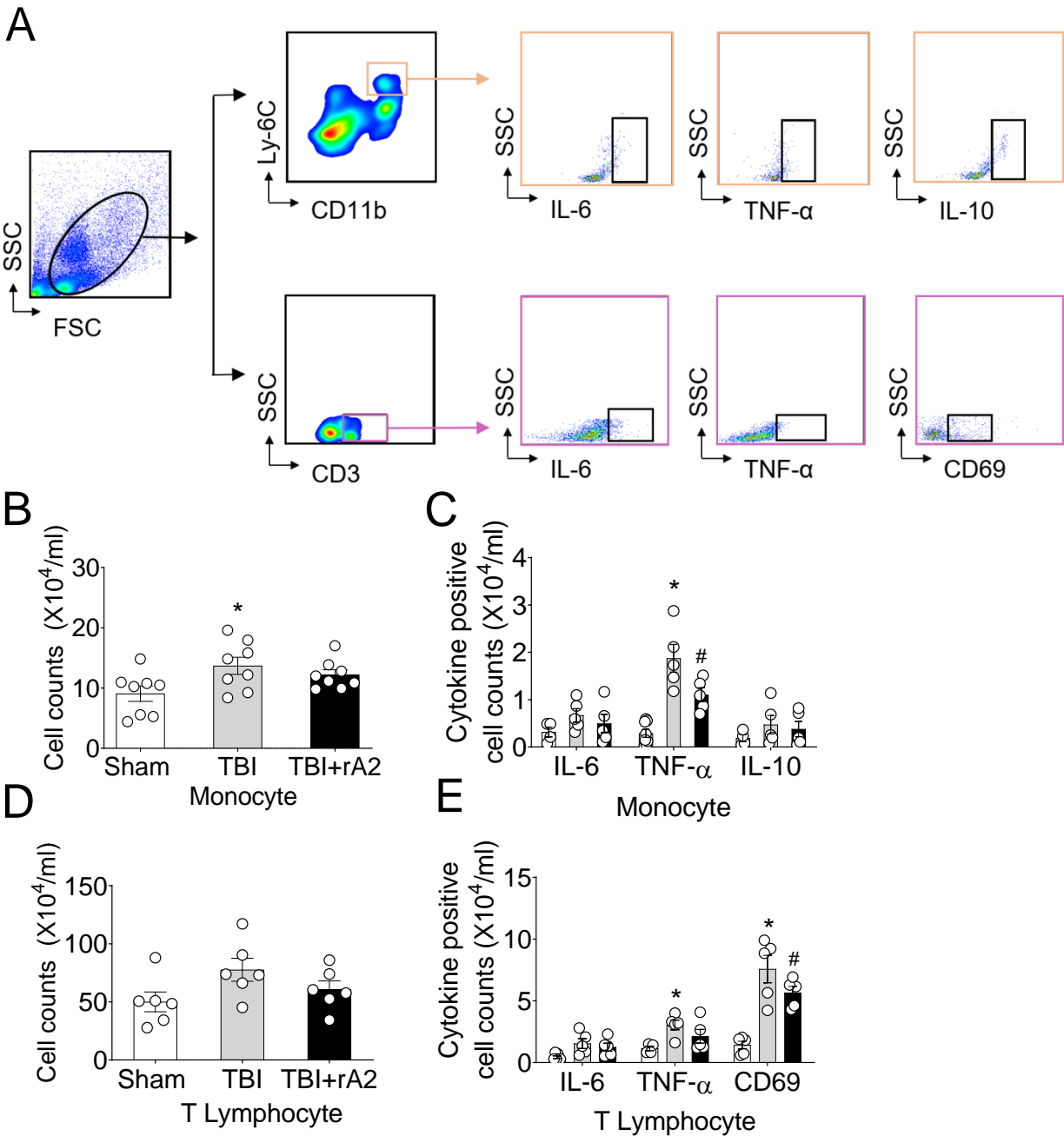

Supplement: Supplementary file 2 — Additional file 2: Supplemental Figure 2. Effect of rA2 on monocytes and T lymphocytes in blood after TBI. (A) Representative gating strategy of peripheral monocytes (CD11b+ Ly6C+), T lymphocytes (CD3+) from single-cell suspensions, and the expression of IL-6, TNF-α, IL-10 in monocytes, and the expression of IL-6, TNF-α, CD69 in T lymphocytes. All gates were set using FMO controls. (B,D) Counts of peripheral monocytes (B) and T lymphocytes (D) of Sham, TBI, and TBI + rA2 mice at 24 h after TBI, n = 6–8. (C) Quantitative analysis shows the expression of IL-6, TNF-α, and IL-10 in monocytes, n = 4–6. (E) Quantitative analysis shows the expression of IL-6, TNF-α, and CD69 in T lymphocytes, n = 4–6. Data are expressed as mean ± SEM, * p < 0.05 compared to sham, # p < 0.05 compared to TBI. [file 12974_2021_2219_MOESM2_ESM.pdf]

# Supplemental Figure 3

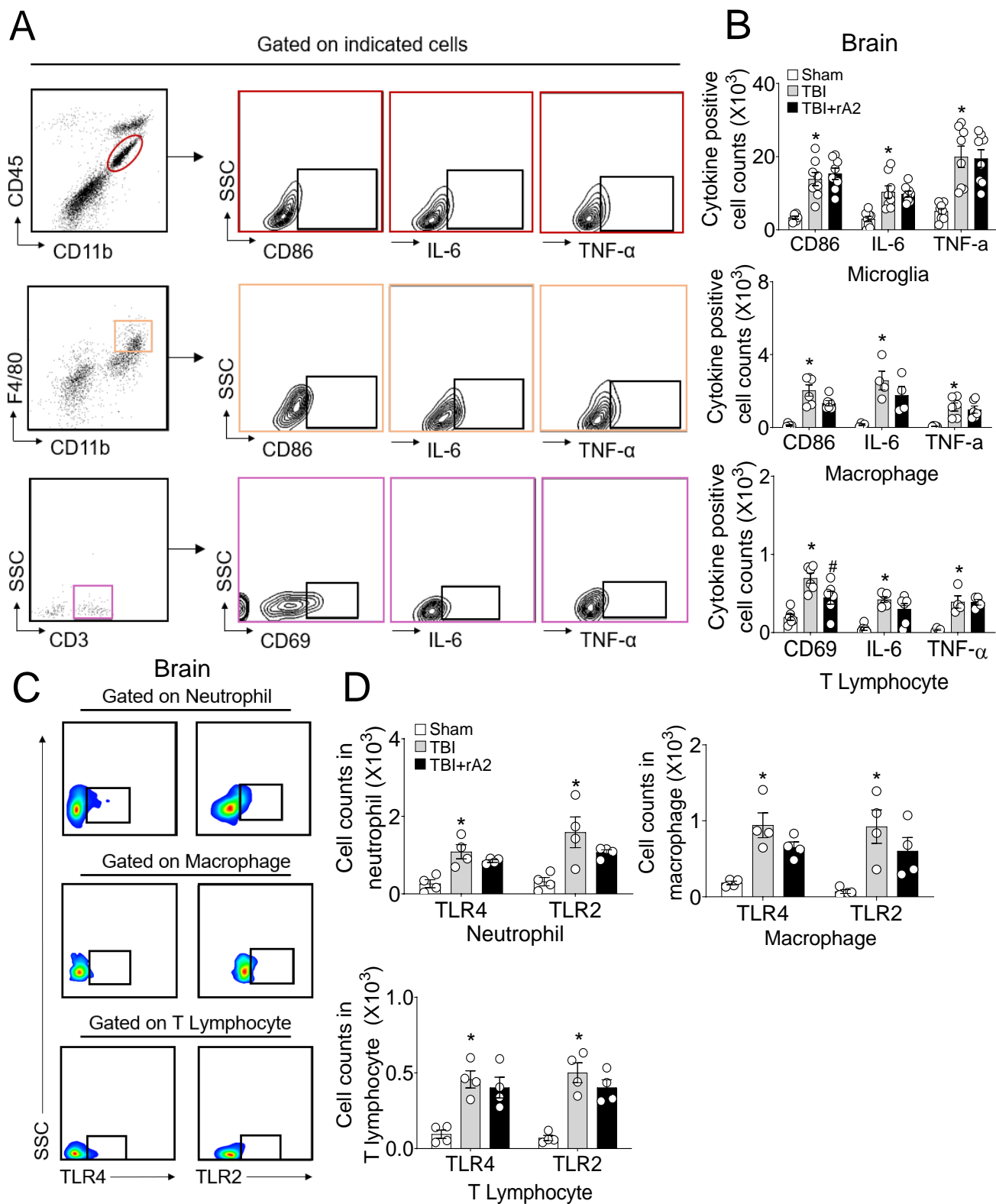

Supplement: Supplementary file 3 — Additional file 3: Supplemental Figure 3. Effect of rA2 on the activation of microglia, macrophage and T lymphocytes in the mouse brain. (A) Representative gating strategy for the expression of CD86, IL-6, TNF-α in microglia (CD11b+CD45int) and macrophages (CD11b+ CD45high F4/80+), and the expression of CD69, IL-6, TNF-α in T lymphocytes (CD45+ CD3+), the brain tissues obtained at 24 h after TBI from Sham, TBI, and TBI + rA2 mice. All gates were set using FMO controls. (B) Cell counts of microglia expressing CD86, IL-6, and TNF-α in the brain, and cell counts of macrophage expressing CD86, IL-6, and TNF-α in the brain, and cell counts of T lymphocyte expressing CD69, IL-6, and TNF-α in brains, n = 4–8. (C) Flow cytometry plots show the expression of TLR4 and TLR2 in brain infiltrated neutrophils, macrophages, and T lymphocytes obtained from brain tissues of TBI at 24 h. (D) Quantitation of TLR4 and TLR2 expressing in brain infiltrated neutrophils, macrophages, and T lymphocytes of Sham, TBI, and TBI + rA2 mice, n = 4. Data are expressed as mean ± SEM, * p < 0.05 compared to Sham, # p < 0.05 compared to TBI. [file 12974_2021_2219_MOESM3_ESM.pdf]
